# Inflammatory myofibroblastic tumor of omentum mimicking an ovarian lesion: A case report

**DOI:** 10.1016/j.radcr.2026.02.021

**Published:** 2026-03-23

**Authors:** Anagha Joshi, Maulik Bhalsod, Sayali Mangal Dhote, Maoulik Kumar Prakashchandra Modi, Navya Mihir Shah

**Affiliations:** a Department of Radiology, Lokmanya Tilak Municipal Medical College and General Hospital, Mumbai, India; b Department of Radiology, The Ottawa Hospital, University of Ottawa, Canada

**Keywords:** Inflammatory myofibroblastic tumor, Omentum, Ovarian mimic, Pediatric, MRI, Case report

## Abstract

Inflammatory myofibroblastic tumor (IMFT) is a neoplastic entity with intermediate malignant potential, characterized by its tendency for local recurrence and, rarely, distant metastasis. It most often occurs in the lung, while extra-pulmonary sites, like the omentum, are rare and present with non-specific clinical and radiologic features, making diagnosis challenging. A 6-year-old girl presented with a 2-month history of intermittent fever, a painless abdominal lump, elevated inflammatory markers, and an ultrasound impression suggestive of an ovarian neoplasm, while magnetic resonance imaging (MRI) showed a right iliac fossa mass separate from the ovary with persistent enhancement. Laparoscopic resection identified the lesion arising from the greater omentum, and histopathology with immunohistochemistry confirmed the diagnosis of IMFT. Omental IMFT is rare and often mimics ovarian or other intra-abdominal tumors, making multimodality imaging, particularly magnetic resonance imaging, crucial for lesion localization and narrowing the differential diagnosis. Although histopathology confirms the diagnosis and complete surgical resection typically yields excellent outcomes, long-term follow-up remains essential due to the risk of recurrence.

## Introduction

Inflammatory myofibroblastic tumor (IMFT) is an uncommon mesenchymal neoplasm characterized by the proliferation of spindle-shaped myofibroblasts admixed with inflammatory cell infiltrates [1,2]. Originally described in the lung by Brunn in 1939 [3], these tumors were initially termed ``inflammatory pseudotumors'' due to their radiologic and clinical resemblance to malignancies [4]. Subsequent cytogenetic studies identified rearrangements involving the anaplastic lymphoma kinase (ALK) gene in more than 50% of IMFT cases, establishing it as a neoplastic entity with intermediate malignant potential, capable of local recurrence and, rarely, distant metastasis [1,[5], [6], [7]].

IMFT may occur in various anatomical locations, most commonly involving the lungs, with the omentum being a rare site of involvement [2,5]. Clinical and imaging findings are often non-specific, frequently mimicking other malignant tumors and posing diagnostic challenges [1,8,9].

We present a case of omental IMFT in a young female initially suspected to have an ovarian neoplasm, and review the literature regarding its imaging characteristics, histopathology, and management.

## Case report

A 6-year-old girl presented with a history of intermittent fever for 2 months and a painless abdominal lump for 1 month. The fever, initially low-grade, temporarily improved with antipyretics and antibiotics prescribed by a local physician but recurred after 1 month, peaking at 104°F and accompanied by palpitations and dizziness, prompting hospital admission. Simultaneously, multiple skin lesions developed over the abdomen and thigh, and her mother noticed a lump in the abdomen.

Initial ultrasonography revealed a 4.4 × 3.3 × 3.0 cm solid, hypoechoic mass in the right iliac fossa. The right ovary was not visualized separately, raising suspicion of a right ovarian neoplastic lesion. Notably, a paternal aunt had a history of hematologic malignancy.

At our center, tumor markers including beta-human chorionic gonadotropin and alpha-fetoprotein were within normal limits, but lactate dehydrogenase was markedly elevated at 990 U/L (normal range: 140-280 U/L). C-reactive protein was 219 mg/L (normal range: <10 mg/L), and D-dimer was 1000 ng/mL (normal range: <250 ng/mL).

**Fig. 1:**
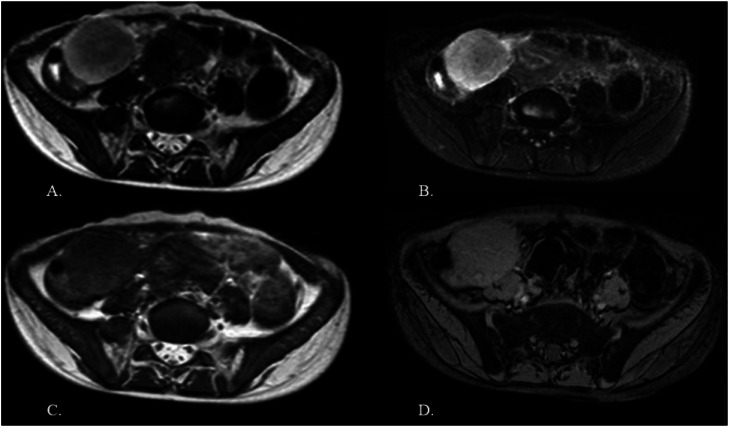
(A and B) T2-weighted and T2-SPAIR images show a well-defined, hyperintense lesion in the right iliac fossa. (C) The T1-weighted image shows the lesion is isointense. (D) No evidence of blooming is noted on the gradient echo sequence.

**Fig. 2:**
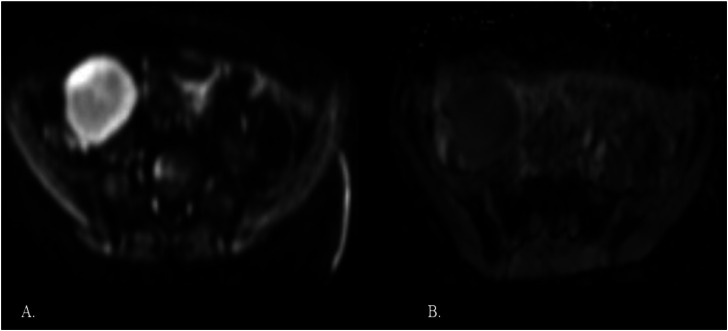
(A and B) The lesions show restricted diffusion with a corresponding low apparent diffusion coefficient value.

**Fig. 3:**
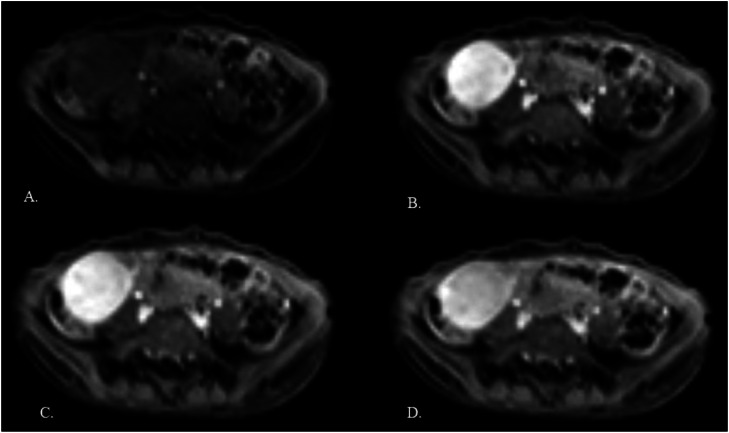
(A-D) Dynamic contrast–enhanced images show intense arterial phase enhancement with persistent enhancement in the venous and delayed phases.

**Fig. 4:**
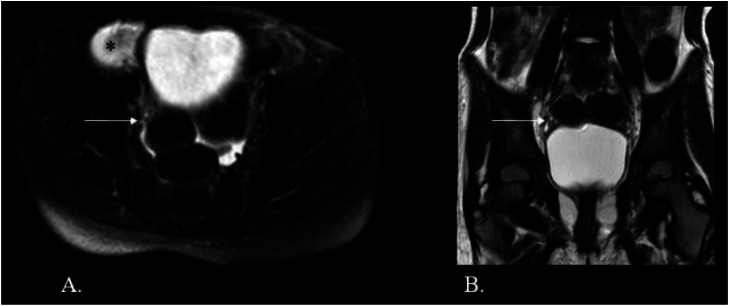
(A and B) Axial T2 SPAIR and coronal T2 images show the right ovary (white arrow) seen separately from the lesion (*).

**Fig. 5:**
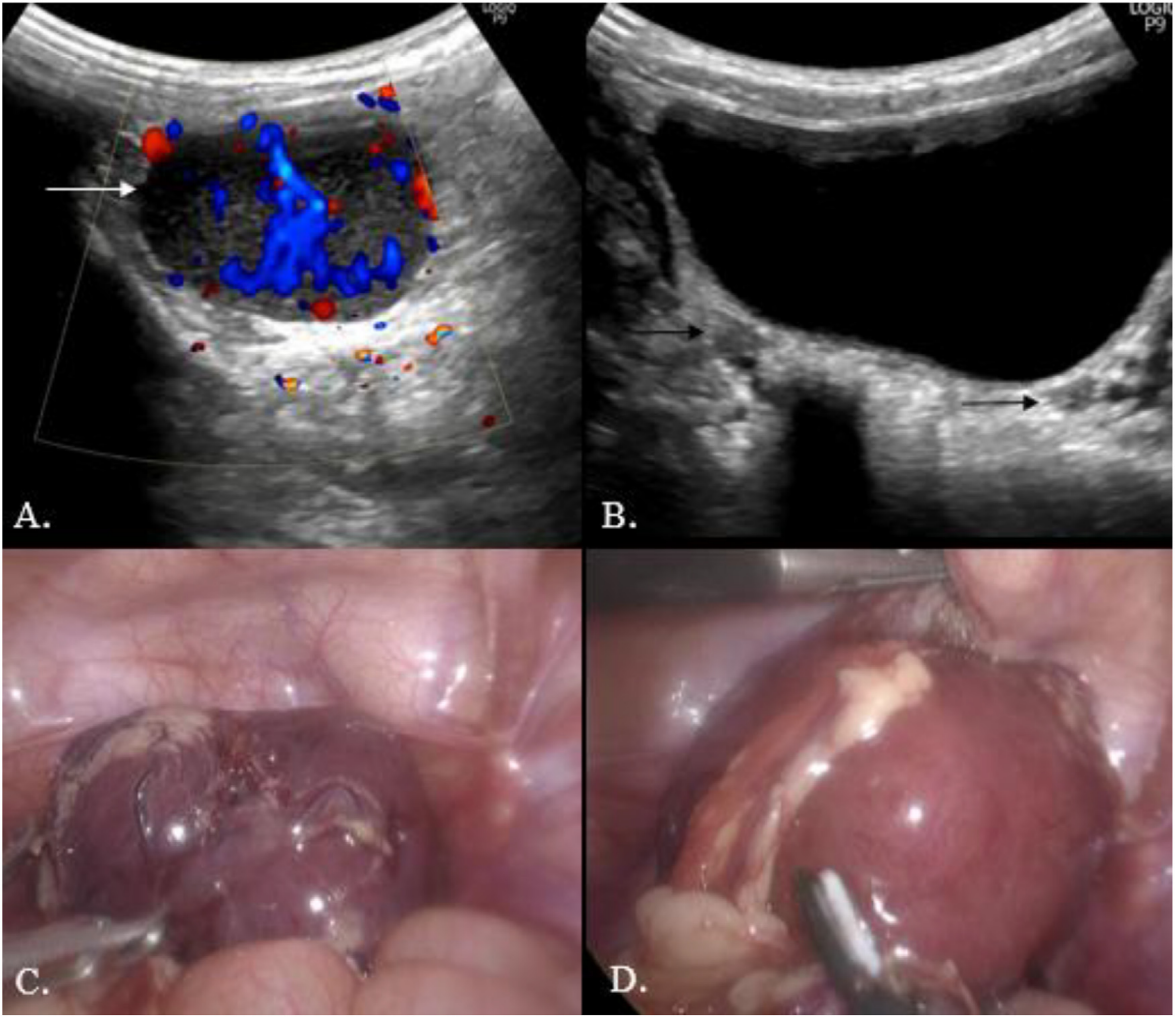
(A) The color Doppler image shows a well-defined hypoechoic lesion (white arrow) with significant internal vascularity. (B) Gray-scale image shows the right and left ovaries (black arrow) (C and D) Intraoperative images show a well-circumscribed lesion with a few vascular channels along the surface of the lesion.

Contrast-enhanced magnetic resonance imaging (MRI) showed a well-defined solid lesion measuring 4.2 × 3.5 × 3.5 cm in the right iliac fossa. It appeared isointense on T1-weighted images and hyperintense on T2-weighted/spectral presaturation attenuated inversion-recovery (SPAIR) images, with restricted diffusion and showing no foci of blooming (Fig. 1, Fig. 2). On dynamic contrast imaging, the lesion showed intense, homogeneous arterial phase enhancement with persistent enhancement on subsequent phases (Fig. 3). It was seen abutting the cecum on the right lateral aspect with effacement of the intervening fat planes. Both ovaries were visualized separately from the lesion (Fig. 4, Fig. 5). No omental or mesenteric fat stranding was noted. Mild ascites was present with a small amount of free fluid in the Pouch of Douglas.

Repeat ultrasound confirmed that the mass was distinct from both ovaries (Fig. 5). Considering the imaging characteristics and inflammatory profile, an omental IMFT was strongly suspected.

The patient underwent laparoscopic resection. Intraoperatively, the mass was located in the greater omentum, separate from both ovaries (Fig. 5). The patient was discharged on postoperative day 7; thereafter, no further fever spikes were observed.

**Fig. 6:**
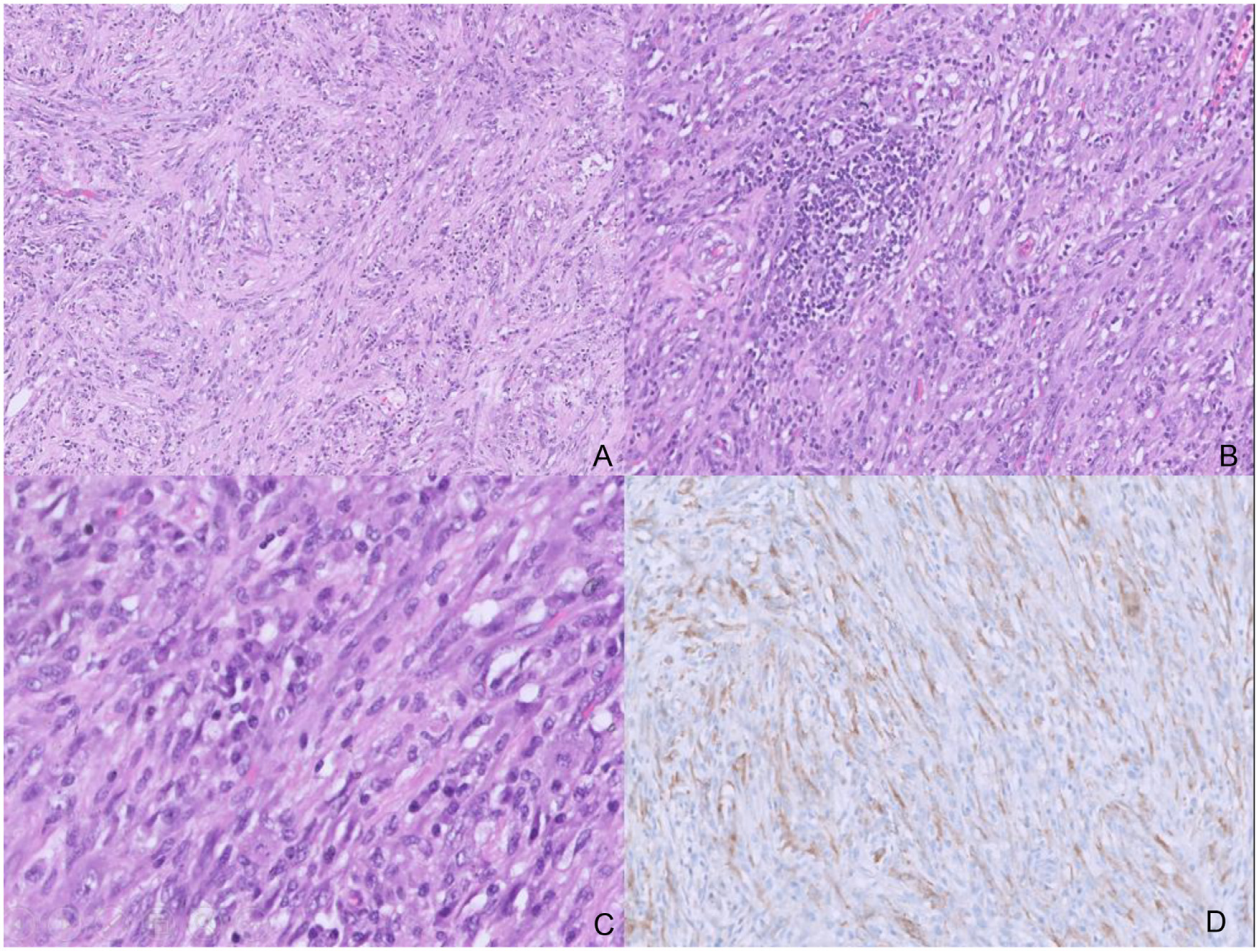
Microscopy and immunohistochemistry slides. (A) Scanner view (H&E;x40) shows a bland spindle cell proliferation with scattered inflammation. (B) Low power view (H&E;x100) shows a cellular spindle cell lesion with abundant dense lymphoplasmacytic inflammation and a lymphoid aggregate. (C) High power view (H&E;x400) shows no atypia in the spindle cells and prominent plasma cells. (D) Immunohistochemistry for ALK shows positive staining in the spindle cells.

The tumor was sent for histopathological examination and immunohistochemistry, which confirmed an IMFT (Fig. 6).

## Discussion

IMFT most commonly affects children and young adults, although it may occur at any age [1,7,10]. The clinical manifestations vary considerably depending on tumor location and size [1,5,11]. Patients may exhibit systemic inflammatory features such as fever, weight loss, malaise, anemia, and elevated inflammatory markers, consistent with our patient’s presentation [1,12]. Omentum as a site of IMFT is extremely rare [13].

Histologically, IMFTs exhibit diverse architectural patterns, ranging from loose myxoid areas populated by spindle cells to densely fibrotic zones with intermixed plasma cells, lymphocytes, and occasional eosinophils [2,14]. Historically, due to this variability, terms such as plasma cell granuloma, xanthogranuloma, and inflammatory fibrosarcoma have been used interchangeably [1,12,14]. A major turning point in the understanding of IMFT came with the discovery of recurrent ALK gene rearrangements at chromosome 2p23, observed in more than 50% of cases [1,6,[15], [16], [17]]. ALK, a tyrosine kinase receptor, when aberrantly activated, contributes to tumor pathogenesis and supports the neoplastic nature of IMFT [16,18]. ALK-positive tumors occur in younger patients, may show higher recurrence rates, and generally have a more favorable prognosis compared with ALK-negative tumors [7,13,19]. Macroscopically, IMFTs typically present as solitary, well-circumscribed, or multilobulated masses with a tan-gray cut surface. While most are firm and solid, some may exhibit calcification, hemorrhage, or central necrosis [1,2].

The imaging findings of an IMFT are often non-specific and variable [9,11,12]. On ultrasonography, these lesions may demonstrate heterogeneous echotexture, with both hypoechoic and hyperechoic lesions described. The margins may be ill-defined or well-defined. Color Doppler often reveals prominent internal vascularity with low-resistance arterial waveforms [1,5,9,[19], [20], [21], [22]]. On computed tomography, IMFTs show variable density depending on lesion composition and may appear hypodense, isodense, or hyperdense compared with adjacent muscles. The enhancement pattern also varies: the lesion might show homogeneous, heterogeneous, or no enhancement. It may show peripheral enhancement in early phases or delayed enhancement in fibrotic regions. Dense central calcifications may be present [1,4,5,20,22]. MRI typically demonstrates low-to-intermediate signal on T1-weighted, heterogeneously high signal on T2-weighted images, and heterogeneous enhancement post contrast. Fibrotic areas appear hypointense on both sequences and show delayed enhancement following contrast administration [1,4,5,19,20]. The lesion margins may be well defined or ill defined, with the latter producing an infiltrative appearance and appearing as highly aggressive malignant lesions [19].

Complete surgical excision with negative margins remains the cornerstone of treatment [1,12,14,19]. Indications of chemotherapy include unresectable cases, incomplete tumor removal, recurrence, or metastasis [5,8,13]. Antibody-mediated therapy, such as ALK inhibitors (eg, crizotinib), has shown promising results [[8], [10], [23]].

## Conclusion

IMFT of the omentum is a rare entity, particularly in children, and may mimic ovarian or other intra-abdominal neoplasms. Multimodality imaging, especially MRI, plays a key role in localizing the lesion and guiding diagnosis. Histopathological confirmation remains crucial. Complete surgical resection typically results in excellent outcomes, though long-term follow-up is advised due to the risk of recurrence.

## Patient consent

Written informed consent was obtained from the patient’s father for the publication of this case report, including all medical data and accompanying images. The patient and her parents were informed that their identity would remain confidential, and all identifying details had been removed to ensure anonymity.

## References

[1] Chung E.M., Biko D.M., Arzamendi A.M., Meldrum J.T., Stocker JT. (2015). Solid tumors of the peritoneum, omentum, and mesentery in children: radiologic-pathologic correlation: from the radiologic pathology archives. Radiographics.

[2] Coffin C.M., Watterson J., Priest J.R., Dehner LP. (1995). Extrapulmonary inflammatory myofibroblastic tumor (inflammatory pseudotumor): a clinicopathologic and immunohistochemical study of 84 cases. Am J Surg Pathol.

[3] Brunn H. (1939). Two interesting benign lung tumors of contradictory histopathology. J Thorac Surg.

[4] Narla L.D., Newman B., Spottswood S.S., Narla S., Kolli R. (2003). Inflammatory pseudotumor. Radiographics.

[5] Liang W., Lin S., Chen Z. (2017). Imaging findings of inflammatory myofibroblastic tumor from the greater omentum: one case report. Medicine (Baltimore).

[6] Griffin C.A., Hawkins A.L., Dvorak C., Henkle C., Ellingham T., Perlman EJ. (1999). Recurrent involvement of 2p23 in inflammatory myofibroblastic tumors. Cancer Res.

[7] Coffin C.M., Hornick J.L., Fletcher CDM. (2007). Inflammatory myofibroblastic tumor: comparison of clinicopathologic, histologic, and immunohistochemical features including ALK expression in atypical and aggressive cases. Am J Surg Pathol.

[8] Siemion K., Reszec-Gielazyn J., Kisluk J., Roszkowiak L., Zak J., Korzynska A. (2022). What do we know about inflammatory myofibroblastic tumours? a systematic review. Adv Med Sci.

[9] Kim S.J., Kim W.S., Cheon J.E., Shin S.M., Youn B.J., Kim I.O. (2009). Inflammatory myofibroblastic tumors of the abdomen as mimickers of malignancy: imaging features in nine children. AJR Am J Roentgenol.

[10] López de Sa A., Pascual A., Garcia Santos J., Mendez R., Bellon M., Ramirez M. (2021). Inflammatory myofibroblastic tumour of an unusual presentation in the uterine cervix: a case report. World J Surg Oncol.

[11] Levy A.D., Shaw J.C., Sobin LH. (2009). Secondary tumors and tumorlike lesions of the peritoneal cavity: imaging features with pathologic correlation. Radiographics.

[12] Kumar U., Thami G., Kamra H., Agarwal N. (2020). Giant omental inflammatory myofibroblastic tumour causing intestinal obstruction: a rare case report and review of literature. Int Surg J.

[13] Karnak I., Senocak M.E., Ciftci A.O., Caglar M., Bingol-Kologlu M., Tanyel F.C. (2001). Inflammatory myofibroblastic tumor in children: diagnosis and treatment. J Pediatr Surg.

[14] Park S.B., Cho K.S., Kim J.K., Lee J.H., Jeong A.K., Kwon W.J. (2008). Inflammatory pseudotumor (myoblastic tumor) of the genitourinary tract. AJR Am J Roentgenol.

[15] Cook J.R., Dehner L.P., Collins M.H., Ma Z., Morris S.W., Coffin CM. (2001). Anaplastic lymphoma kinase (ALK) expression in the inflammatory myofibroblastic tumor: a comparative immunohistochemical study. Am J Surg Pathol.

[16] Coffin C.M., Patel A., Perkins S., Elenitoba-Johnson K.S., Perlman E., Griffin CA. (2001). ALK1 and p80 expression and chromosomal rearrangements involving 2p23 in inflammatory myofibroblastic tumor. Mod Pathol.

[17] Minoo P., Wang HY. (2012). ALK-immunoreactive neoplasms. Int J Clin Exp Pathol.

[18] Gleason B.C., Hornick JL. (2008). Inflammatory myofibroblastic tumours: where are we now?. J Clin Pathol.

[19] Rasalkar D.D., Chu W.C., To K.F., Cheng F.W., Li CK. (2010). Radiological appearance of inflammatory myofibroblastic tumour. Pediatr Blood Cancer.

[20] Sedlic T., Scali E.P., Lee W.K., Verma S., Chang SD. (2014). Inflammatory pseudotumours in the abdomen and pelvis: a pictorial essay. Can Assoc Radiol J.

[21] Uysal S., Tuncbilek I., Unlubay D., Tiras U., Bilaloglu P., Kosar U. (2005). Inflammatory pseudotumor of the sigmoid colon mesentery: US and CT findings. Eur Radiol.

[22] Patnana M., Sevrukov A.B., Elsayes K.M., Viswanathan C., Lubner M., Menias CO. (2012). Inflammatory pseudotumor: the great mimicker. AJR Am J Roentgenol.

[23] Butrynski J.E., D'Adamo D.R., Hornick J.L., Dal Cin P., Antonescu C.R., Jhanwar S.C. (2010). Crizotinib in ALK-rearranged inflammatory myofibroblastic tumor. N Engl J Med.

